# 437. Does “Long COVID” Resolve by One Year in U.S. Military Health System Beneficiaries?

**DOI:** 10.1093/ofid/ofad500.507

**Published:** 2023-11-27

**Authors:** Stephanie A Richard, Celia Byrne, Jennifer Rusiecki, Catherine Berjohn, Tahaniyat Lalani, Alfred Smith, Rupal Mody, Anuradha Ganesan, Rhonda Colombo, David Lindholm, Michael Morris, Nikhil Huprikar, Christopher Colombo, Christina Schofield, Milissa U Jones, Katrin Mende, David Saunders, Jeffrey Livezey, David Chang, Evan Ewers, Carlos Maldonado, Ann Scher, Anthony C Fries, Ryan C Maves, Nusrat J Epsi, Kat Schmidt, Margaret Sanchez Edwards, Mark Simons, David R Tribble, David R Tribble, Robert O’Connell, Brian Agan, Timothy Burgess, Simon Pollett

**Affiliations:** Infectious Disease Clinical Research Program, Department of Preventive Medicine and Biostatistics, Uniformed Services University of the Health Sciences, Bethesda, MD, USA, Bethesda, Maryland; Uniformed Services University of the Health Sciences, Bethesda, Maryland; Uniformed Services University of the Health Sciences, Bethesda, Maryland; Naval Medical Center San Diego, San Diego, California; Naval Medical Center Portsmouth, Portsmouth, Virginia; Naval Medical Center, Portsmouth, Virginia; William Beaumont Army Medical Center, El Paso, Texas; Infectious Disease Clinical Research Program, USUHS; Henry M. Jackson Foundation for the Advancement of Military Medicine Inc, Bethesda, Maryland; Infectious Disease Clinical Research Program, USUHS, Tacoma, Washington; Department of Medicine, Uniformed Services University of the Health Sciences; Brooke Army Medical Center, San Antonio, Texas; Brooke Army Medical Center, Fort Sam Houston, Texas; Walter Reed National Military Medical Center, Bethesda, Maryland; Madigan Army Medical Center, Tacoma, Washington; Madigan Army Medical Center, Tacoma, Washington; Uniformed Services University, Bethesda, Maryland; Brooke Army Medical Center, Fort Sam Houston, Texas; Uniformed Services University of the Health Sciences, Bethesda, MD, USA, Bethesda, Maryland; USUHS, Bethesda, Maryland; Fort Belvoir Community Hospital, Fort Belvoir, Virginia; Fort Belvoir Community Hospital, Fort Belvoir, Virginia; Womack Army Medical Center, Fort Bragg, North Carolina; Uniformed Services University of the Health Sciences, Bethesda, Maryland; U.S. Air Force School of Aerospace Medicine, Dayton, Ohio; Wake Forest University School of Medicine, Winston-Salem, North Carolina; Uniformed Services University of the Health Sciences, Bethesda, Maryland; Infectious Disease Clinical Research Program, USUHS, Tacoma, Washington; Infectious Disease Clinical Research Program, Department of Preventive Medicine and Biostatistics, Uniformed Services University of the Health SciencesHenry M. Jackson Foundation for the Advancement of Military Medicine, Bethesda, Maryland; USUHS, Bethesda, Maryland; Uniformed Services University of the Health Sciences, Bethesda, Maryland; Uniformed Services University of the Health Sciences, Bethesda, Maryland; Infectious Disease Clinical Research Program, USUHS, Tacoma, Washington; Infectious Disease Clinical Research Program, Department of Preventive Medicine and Biostatistics, Uniformed Services University of the Health Sciences, Bethesda, MD, USA, Bethesda, Maryland; USUHS, Bethesda, Maryland; Infectious Disease Clinical Research Program, Department of Preventive Medicine and Biostatistics, Uniformed Services University of the Health Sciences, Bethesda, MD, USA, Bethesda, Maryland

## Abstract

**Background:**

The long-term duration of post COVID condition (PCC, “Long COVID”) remains unclear. In this study, we estimated the risk of healthcare encounters in Military Health System (MHS) beneficiaries for the 12 months post SARS-CoV-2 diagnosis, adjusting for prior healthcare use, and compared to those without known prior SARS-COV-2 diagnosis.

**Methods:**

Follow up continues for the Epidemiology, Immunology and Clinical Characteristics of Emerging Infectious Diseases of Pandemic Potential (EPICC) COVID-19 cohort study MHS beneficiaries who were tested for SARS-COV-2 or vaccinated from March 2020 to April 2022. Participants with SARS-COV-2 diagnosis from 3/1/20 through 12/31/21 were matched 1:1 with participants in the same age group with no record of SARS-COV-2 diagnosis. We identified categories of ICD-10 diagnoses occurring from 3 months before through 12 months after first SARS-COV-2 diagnosis (or matched time point) from electronic medical records. Multivariable Poisson regression models were used to estimate the risk of ICD-10 diagnosis categories for those with past SARS-COV-2 diagnosis, compared to those with no SARS-COV-2 diagnosis, adjusting for age, sex, BMI, variant era, and prior healthcare use.

**Table 1.**
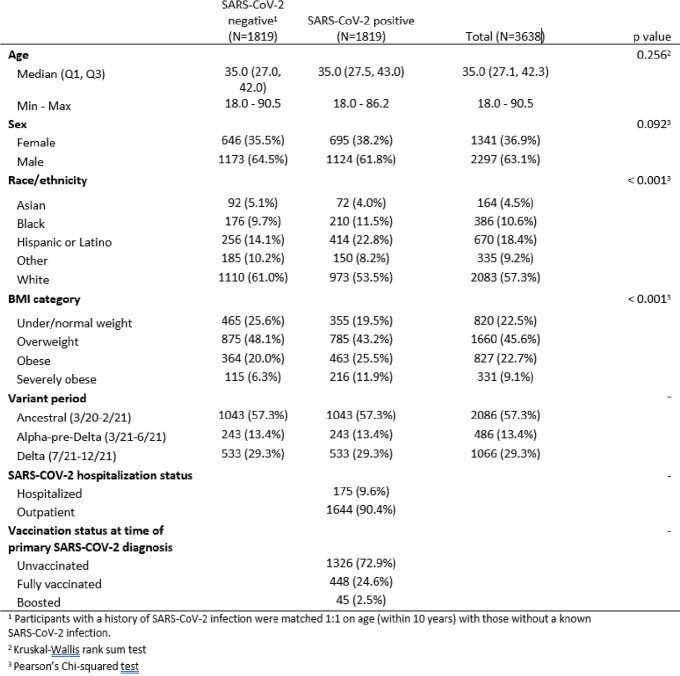
Characteristics of matched Epidemiology, Immunology and Clinical Characteristics of Emerging Infectious Diseases of Pandemic Potential (EPICC) participants included in analyses. Statistical comparisons are Pearson’s Chi squared tests, unless otherwise specified.

**Results:**

Analyses included 1,819 matched pairs with a median age of 35 years. Participants were primarily male (63.0%) or white (57.3%) (Table 1) and severe acute COVID-19 was infrequent (9.6% hospitalized). Compared to those without a history of SARS-COV-2 diagnosis, medical encounters for all diagnosis groups (pulmonary, cardiovascular, diabetes, anxiety/depression, and neurology) were elevated in the first month after SARS-COV-2 diagnosis (Figures 1 and 2). Among the different diagnosis categories, only pulmonary diagnoses remained elevated at 9 months post-infection compared to those without a history of SARS-COV-2 diagnosis (risk ratio: 1.95 (95% CI 1.34, 2.83)).

Percent of EPICC participants with medical encounters / diagnoses (by organ system or other domain) in health records. Participants without a history of SARS-CoV-2 infection were assigned the infection date of their matched case.

**Figure:**
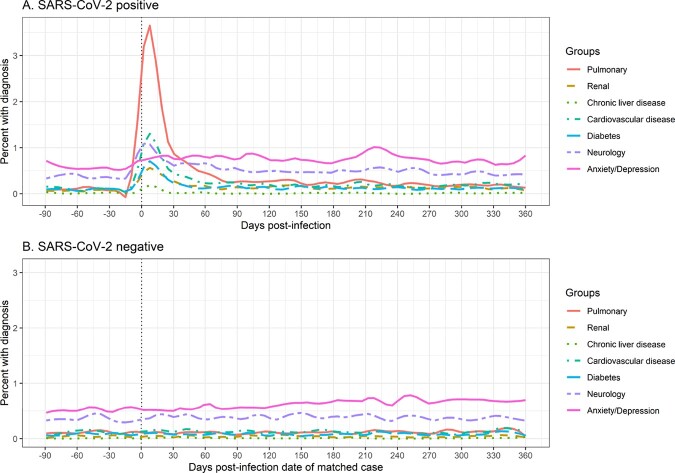

Poisson regression analysis run using each category of healthcare encounters (pulmonary, cardiovascular, diabetes, neurology, and anxiety/depression) as the outcome. The models included time in 30-day periods around SARS-CoV-2 diagnosis date (or matched time point), SARS-CoV-2 diagnosis status, age, sex, BMI category, and variant/calendar period (calendar times with predominant Ancestral, Alpha, or Delta circulation), as well as a random effect for participant.

**Figure:**
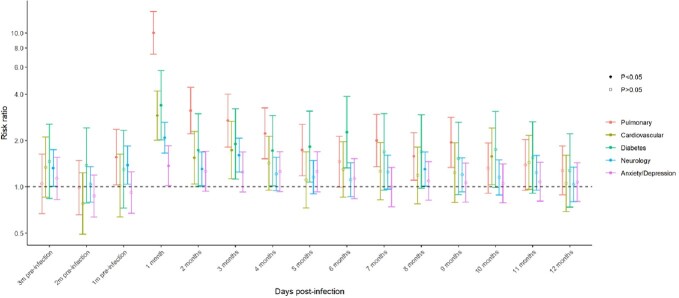

**Conclusion:**

MHS beneficiaries with prior SARS-COV-2 diagnosis were at higher risk of pulmonary-associated healthcare encounters through 9 months post-infection compared to those without prior SARS-COV-2 diagnosis, even after adjusting for baseline characteristics and calendar time. Future work will assess the effect of vaccination and boosting on this relationship.

**Disclosures:**

**Michael Morris, MD**, Janssen Pharmaceuticals: Paid speaker (unrelated to this project and COVID-19 in general) **Ryan C. Maves, MD**, Sound Pharmaceuticals: Grant/Research Support **Mark Simons, PhD**, AstraZeneca: TBD **Timothy Burgess, MD**, AstraZeneca: TBD **Simon Pollett, MBBS**, AstraZeneca: The IDCRP and the Henry M. Jackson Foundation (HJF) were funded to conduct an unrelated phase III COVID-19 monoclonal antibody immunoprophylaxis trial

